# The effectiveness of battlefield acupuncture in addition to standard physical therapy treatment after shoulder surgery: a protocol for a randomized clinical trial

**DOI:** 10.1186/s13063-020-04909-8

**Published:** 2020-12-03

**Authors:** Michael S. Crowell, Richard A. Brindle, John S. Mason, Will Pitt, Erin M. Miller, Matthew A. Posner, Kenneth L. Cameron, Donald L. Goss

**Affiliations:** 1 Baylor University - Keller Army Community Hospital Division 1 Sports Physical Therapy Fellowship, West Point, NY USA; 2 John A Feagin, Jr. Sports Medicine Fellowship, Keller Army Community Hospital, West Point, NY USA; 3 Department of Physical Therapy, High Point University, High Point, NC USA

**Keywords:** Battlefield acupuncture, Opioids, Post-surgical pain

## Abstract

**Introduction:**

There is a large incidence of shoulder instability among active young athletes and military personnel. Shoulder stabilization surgery is the commonly employed intervention for treating individuals with instability. Following surgery, a substantial proportion of individuals experience acute post-operative pain, which is usually managed with opioid pain medications. Unfortunately, the extended use of opioid medications can have adverse effects that impair function and reduce military operational readiness, but there are currently few alternatives. However, battlefield acupuncture (BFA) is a minimally invasive therapy demonstrating promise as a non-pharmaceutical intervention for managing acute post-operative pain.

**Methods:**

This is a parallel, two-arm, single-blind randomized clinical trial. The two independent variables are intervention (2 levels, standard physical therapy and standard physical therapy plus battlefield acupuncture) and time (5 levels, 24 h, 48 h, 72 h, 1 week, and 4 weeks post shoulder stabilization surgery). The primary dependent variables are worst and average pain as measured on the visual analog scale. Secondary outcomes include medication usage, Profile of Mood States, and Global Rating of Change.

**Discussion:**

The magnitude of the effect of BFA is uncertain; current studies report confidence intervals of between-group differences that include minimal clinically important differences between intervention and control groups. The results of this study may help determine if BFA is an effective adjunct to physical therapy in reducing pain and opioid usage in acute pain conditions.

**Trial registration:**

ClinicalTrials.gov NCT04094246. Registered on 16 September 2019.

## Background

Shoulder and glenohumeral instability are serious problems for athletes and military personnel [1–6]. In general, younger and more active populations are at a high risk for glenohumeral instability [2]. However, military personnel sustain shoulder dislocations at a greater rate compared to the overall United States (U.S.) population (3.13 per 1000 person years) [2, 7, 8]. The risk of shoulder dislocation is greater still at Military Service Academies with an incidence of 4.35 per 1000 person years [5]. Given the highly physical demands of competitive athletics and military service, investigations of shoulder dislocation prevention and rehabilitation are warranted.

Management of shoulder dislocations usually includes shoulder stabilization surgery, which is considered the “gold standard” for treatment of shoulder dislocation in young, active patients [4, 9–11]. This type of orthopedic surgery is associated with excellent short-term results and good long-term maintenance of shoulder function after chronic, unidirectional, and traumatic dislocation [4, 9–11]. However, as many as 80% of patients who undergo orthopedic surgery experience acute post-operative pain, which acutely impairs physical function and is a risk factor for the development of persistent pain [12]. Prolonged pain and physical impairment negatively affects military careers and impacts military readiness.

Physical therapy interventions focus on reducing pain and physical impairment associated with shoulder surgery. Standard care for patients post-shoulder surgery consists of two phases: (1) early rehabilitation from 24 h to 2 weeks post-surgery and (2) mid-term rehabilitation from 2 to 6 weeks post-surgery. Early rehabilitation after shoulder stabilization surgery focuses on pain control, protection of the surgical repair, prevention of a “stiff” shoulder, and regaining scapular control [13–15]. Within 6 weeks post-operative, the patient should achieve a minimum of 90° of shoulder elevation and 10–20° of external rotation at 50° of scaption [13–15]. Elevated levels of pain may contribute to an inability to regain range of motion during early rehabilitation after shoulder stabilization surgery.

Opioid prescription is the widely employed method to manage post-operative pain in both Civilian and Military Health Systems. In representative civilian populations, 26% of patients undergoing arthroscopic shoulder procedures received at least one refill of their opioid prescription following surgery compared to 12% and 13% of patients following hip and knee procedures, respectively [16]. The Veteran’s Affairs (VA) administration reported the average length of post-operative opioid use in all post-surgical patients as 15 days [17], which may be longer for shoulder post-surgical patients. Prolonged use of opioids post-surgery presents a possible risk for dependence and side effects such as drowsiness and impaired cognition, leading to necessary duty limitations that affect operational readiness. However, currently, there are few alternative pain management options. Thus, alternative and/or integrative methods of treating acute and chronic post-surgery pain without readiness reducing side-effects are needed.

Battlefield acupuncture (BFA), an auricular acupuncture protocol developed to treat acute and chronic pain in austere environments [18], may provide an integrative pain treatment option to decrease prescription medication usage for a myriad of musculoskeletal conditions [19–22]. The mechanisms of pain reduction by BFA are not fully understood. Chemical mechanisms for the treatment’s effectiveness have been suggested and include endogenous opioid release and the inhibition of neurotransmitters [23, 24]. Multiple structures in the brain are responsible for processing and modulating painful stimuli to include the thalamus and sensory cortex. Limited research using functional magnetic resonance imaging and positive electron tomography has shown auricular acupuncture attenuates pain sensation within the sensory cortex, thereby reducing the perception of pain [25].

BFA has been taught and adopted throughout all branches of the Department of Defense (DoD) over the past two decades to provide alternatives to side effect laden prescription medications [22]. Despite wide-scale adoption by military medical providers, there is inconsistent evidence of BFA’s effectiveness in reducing pain and opioid medication use. In a recent systematic review, pooled results indicated treatment of pain with auricular acupuncture had greater self-reported reductions in pain compared to sham interventions [21]. Studies of BFA in addition to standard care reported promising but inconsistent reductions in short-term pain. Patients with low back pain reported a greater reduction in pain associated with standard treatment supplemented with BFA compared to standard treatment alone [19]. However, following lower extremity surgery patients reported similar pain, opioid use, and quality of life with BFA plus standard care compared with standard of care alone or placebo treatments [26]. Most recently, BFA supplemented with standard care resulted in great pain reduction throughout the first week after shoulder surgery compared to standard care alone in a small cohort of U.S. Military Academy Cadets [27]. However, these findings were limited due to the small sample size and wide confidence intervals. More evidence is required to better understand the effectiveness of BFA to reduce pain in military populations.

BFA is an integrative pain treatment method that may be effective in reducing pain and opioid use post-surgery. The purpose of this study is to determine differences in pain, mood, self-reported improvement, and medication use during and after a standard physical therapy rehabilitation protocol supplemented with BFA, compared to a standard physical therapy rehabilitation protocol alone, for patients following shoulder stabilization surgery. The primary objective is to assess the effect of BFA on post-surgical pain (average and worst pain at 48 h and 72 h post-surgery). We hypothesize standard rehabilitation supplemented with BFA will produce greater reductions in pain compared to standard rehabilitation alone at 48 h and 72 h post-surgery.

Additional objectives are to assess the effect of BFA on: (1) medication use, (2) mood, and (3) self-reported improvement throughout the 4-week post-operative rehabilitation period, and (4) pain at 1 week and 4 weeks post-surgery. It is hypothesized that participants receiving standard rehabilitation supplemented with BFA will have greater reductions in medication use and improvements in patient’s self-reported mood and improvement across the 4-week post-operative follow-up period. Further, participants receiving standard rehabilitation with BFA will have lower pain levels at 1 and 4 weeks post-surgery compared to those receiving standard rehabilitation only.

## Methods/design

### Trial design

**Fig. 1:**
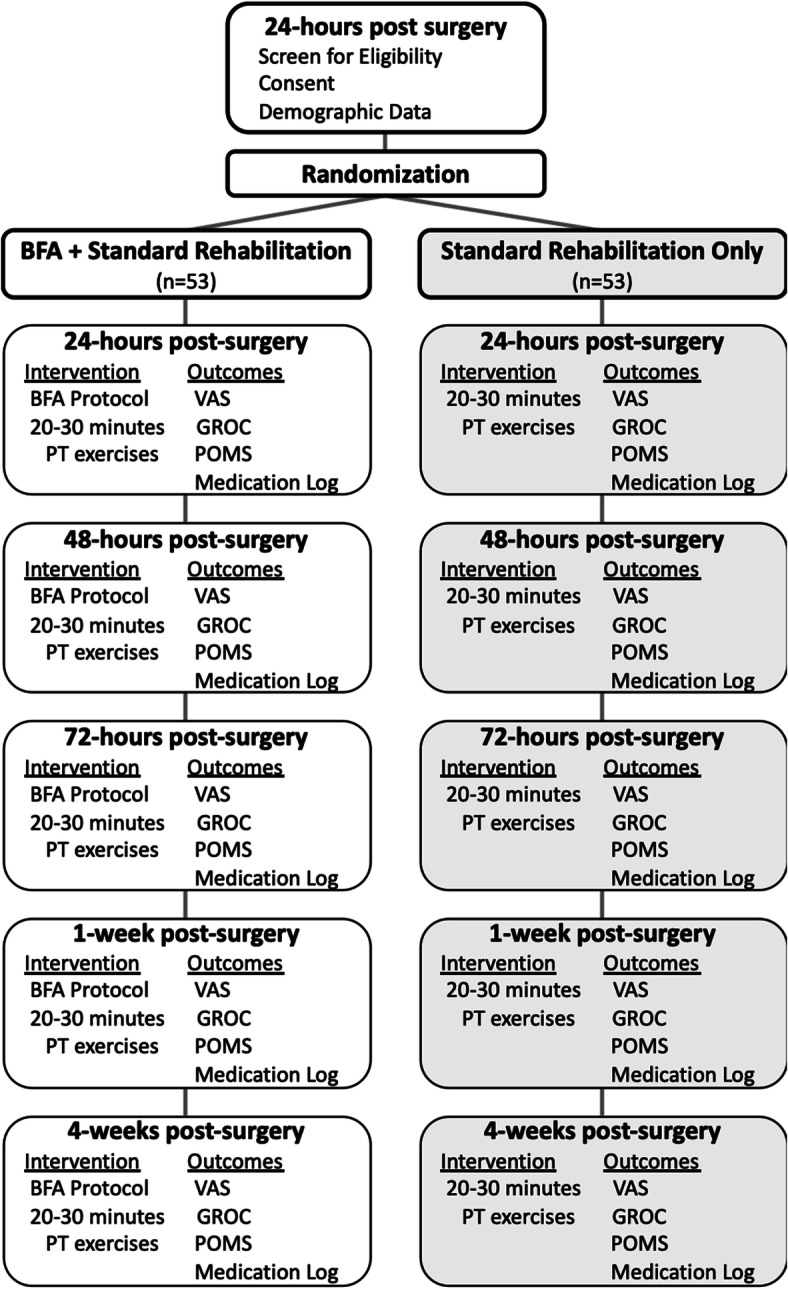
Proposed recruitment flow for the study along with interventions performed and outcomes collected at each post-surgical timepoint. BFA, battlefield acupuncture; VAS, visual analog scale; POMS, Profile of Mood States; GROC, Global Rating of Change Scale

This study is a parallel, two-arm single-blind randomized clinical trial. All participants will complete five study sessions following surgery: 24 h, 48 h, 72 h, 1 week, and 4 weeks post-surgery (Fig. 1). Participants will receive an email the day prior to each study session to promote retention and compliance. Data collection began in November 2019 and will continue for 4 years. All components of the study will be completed at the U.S. Military Academy at West Point, NY. The current Standard Protocol Items: Recommended for Interventional Trials (SPIRIT) guidelines for creating protocols for randomized clinical trials were followed (Supplemental Materials) [28]. Results of this trial will be reported in accordance with Consolidated Standards of Reporting Trials (CONSORT) Statement [29] and Template for Intervention Description and Replication (TIDieR) Checklist [30].

### Participants and study setting

Participants will be recruited from the population of patients presenting to the Arvin Cadet Physical Therapy Clinic and the Keller Army Community Hospital (KACH) Physical Therapy and Orthopedic Clinics prior to and status-post shoulder stabilization surgery. A total of 105 male and female Department of Defense (DoD) beneficiaries, ages 17–55 will be recruited for the study. A 15% drop-out rate is anticipated, which will result in at least 90 patients completing the study, with 45 patients per treatment group. On average KACH orthopedic surgeons perform eight shoulder stabilization surgeries monthly, suggesting the recruitment goal is feasible.

Inclusion criteria:

1. DoD beneficiaries age 18 to 55 years old (17 if cadet)
2. Prior to or within 24 h post shoulder stabilization surgery
3. Self-reported pain rating of at least 2 out of 10 on a Numerical Pain Rating Scale (NPRS)

Exclusion criteria:

1. Self-reported pregnancy
2. History of bloodborne pathogens, infectious disease, or active infection
3. History of metal allergy
4. History of bleeding disorders or currently taking anti-coagulant medications
5. Participants not fluent in English

### Randomization/allocation/blinding

Participants will be screened prior to consent as part of routine clinical care by the investigative team during the first post-operative visit, 24 h after surgery. Those meeting criteria for inclusion will be informed as to the need and purpose of the research and invited to participate. Study participants will complete informed consent followed by a baseline examination (Supplemental Materials). Consenting participants will be randomized into one of two groups via a concealed allocation process: the control group (standard physical therapy rehabilitation) or the intervention group (BFA plus a standard physical therapy rehabilitation). Following informed consent and baseline examination, a second investigator blinded to the baseline examination will open a sealed envelope containing a folded index card labeled with the participant’s group assignment. An investigator not involved with participant recruitment or data collection will create the randomization sequence with a 1:1 allocation using a random permuted block approach on Excel 2010 (Microsoft, Redmond, WA, USA) [31]. The random permuted block approach was utilized to keep intervention arms relatively equivalent throughout the data collection process. Group assignment will be recorded with a unique participant identifier and secured in a separate folder until completion of all data collection through the final follow-up.

Participants and the treating physical therapists will not be blinded to group assignment. Verification of the medication log by the outcome assessor may be another source of bias. Outcome assessors who record pain, self-report function, and mood surveys, perform data reduction, and perform data analysis will be blinded to the participants’ treatment group. Participants frequently require clarification of outcome measure instructions during completion of data collection forms and may require additional assistance if the surgery is performed on their dominant extremity. Participants will interact with the outcome assessor and complete data collection forms behind a closed curtain, where the outcome assessor is not able to see the patient. Non-standard blinding techniques are often implemented in trials assessing nonpharmacological treatments and may minimize either conscious or unconscious researcher recording and reporting bias during completion and verification of the data collection forms [32].

### Interventions

#### Standard physical therapy rehabilitation (active control group)

Both groups will receive standard post-surgical physical therapy according to guidelines developed at our institution (Additional file 2). Five physical therapy sessions, approximately 30 min in duration, will occur at the 24-h initial time point and at the 48-h, 72-h, 1-week, and 4-week follow-up visits. During each visit, the physical therapist will expose the surgical site to check for any signs of infection and review the post-operative precautions with the patient. The patient will perform range of motion and muscle activation exercises consisting of modified pendulum exercises, active range of motion exercises for the elbow/wrist/hand, active-assisted shoulder flexion and external rotation, gentle isometric muscle activation of the rotator cuff and deltoid, and scapular muscle activation exercises. Ice and intermittent compression will be applied for control of pain and swelling, as needed. Patients utilizing these rehabilitation guidelines after shoulder stabilization surgery have demonstrated significant improvements in pain, range of motion, and function that meet established goals for progression to the next phase of rehabilitation [33].

#### Battlefield acupuncture (study intervention group)

**Fig. 2:**
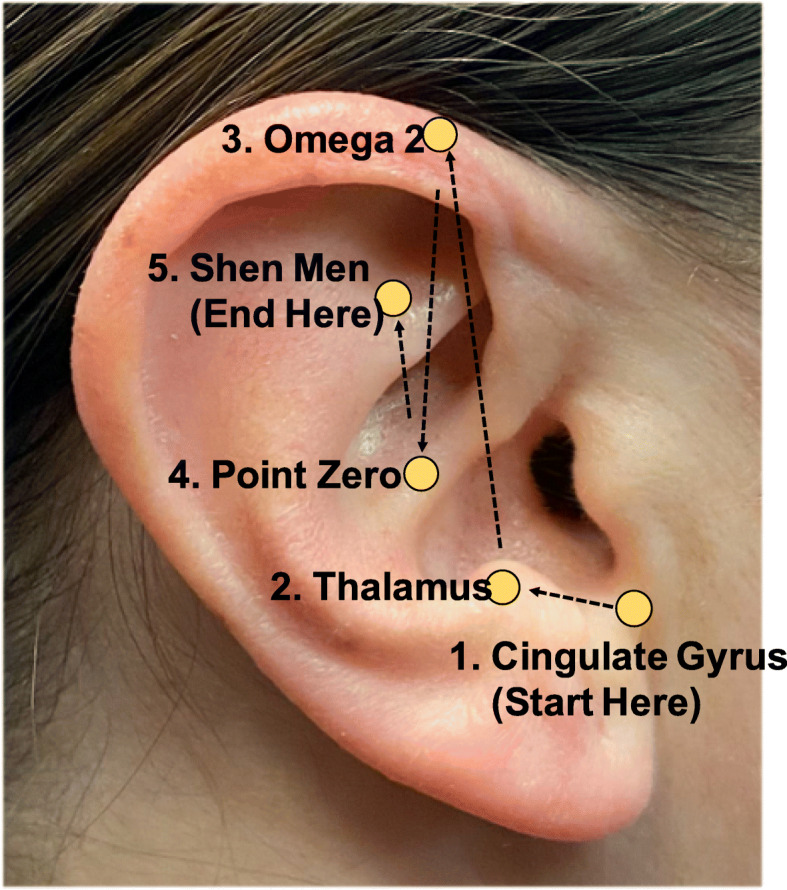
The five auricular acupuncture points of the battlefield acupuncture protocol

Using aseptic technique (proper handwashing, personal protective equipment (PPE), and ear cleansing with an alcohol swab), auricular acupuncture using the five points specified within the BFA protocol will be administered. Each ear will potentially be punctured with ASP needles at five sequential points: cingulate gyrus, thalamus, omega 2, point zero, and Shen-Men (Fig. 2, Additional file 3). The acupuncture sequence will begin on the same side of the shoulder surgery (ipsilateral ear) and begin at the cingulate gyrus point. Following each ASP needle placement, the participant will be asked to stand and move/walk for at least 30 s while being monitored for any side effects, including light-headedness, dizziness/loss of balance or nausea. Additionally, self-reported current pain level will be reassessed. If the participant’s pain is above zero to one out of 10 on the NPRS, the contralateral ear will be punctured with the ASP needle in the cingulate gyrus. ASP needle application will continue, alternating between ipsilateral and contralateral ears in order through the remaining four points, until the desired pain level of zero to one out of 10 is achieved or until all 10 ASP needles are placed.

There is no standard time for the ASP needles to remain in the participant’s ears, but they may remain up to 3–5 days as they naturally work their way out of the skin. Participants will be instructed on how to properly care for and remove the ASP needles should they become irritating. There are no documented cases of loss of treatment effect should participants remove ASP needles prematurely to their natural falling out. All participants in the study intervention group will receive BFA treatment at the initial (24-h) time point. Repeat treatment intervention may be provided during the 48-h, 72-h, and 1-week follow-up visits, but will not coincide with standard physical therapy rehabilitation sessions. The decision whether BFA intervention will be provided during the follow-up visits of the intervention group participants will be left to the discretion of the treating physical therapist and preference of the patient. Treating physical therapists will take into account the patient’s current pain level and prior response to BFA. Additionally, for each participant, the number of BFA interventions executed, the number of ASP needles inserted at each intervention, and the location of each needle’s placement will be recorded for descriptive analyses. If ASP needles remain in place at follow-up visits and the patient requests additional treatment, additional ASP needles will be placed adjacent to locations used previously.

All BFA treatments will be performed by investigators trained and certified on the standard BFA protocol developed by Dr. Niemtzow [18]. Participants in the intervention group will continue to receive the standard of care in accordance with the post-operative protocol between study follow-up visits.

### Outcome measures

Demographic characteristics will be recorded and include sex, age, ethnicity, military demographics, height, weight, and surgical history. The primary outcome measure will be pain (average and worst pain over the past 24 h, assessed on the visual analog scales [VAS]) at 24 h, 48 h, 72 h, 1 week, and 4 weeks after surgery. Two previous studies suggest that the greatest effect of BFA on pain occurs at 1 week or less [27, 34]. Secondary outcome measures will be medication use (opioids and non-opioid medications), patient self-reported mood (Profile of Mood States [POMS]), and patient self-reported improvement (Global Rating of Change Scale [GROC]). All participants will complete all outcome measures at five post-surgical timepoints: 24 h, 48 h, 72 h, 1 week, and 4 weeks (Fig. 1).

The VAS assesses the perception of pain intensity by asking the patient to mark their level of pain along a 100-mm line, where the left limit indicates no pain and the right limit indicates the worst pain imaginable [35, 36]. The VAS is a valid and reliable measure of pain intensity [35–39] with a minimal clinically important difference (MCID) of 10 mm and patient acceptable symptoms state of 30 mm in acute, post-operative pain [38].

The POMS is a 40-item questionnaire designed to measure the transient emotional states of tension-anxiety, depression-dejection, fatigue-inertia, vigor-activity, confusion-bewilderment, and anger-hostility in sports and other settings [40]. The POMS is a valid and reliable measure of mood in athletes and sports [40, 41].

The GROC is a 15-point self-report Likert scale (− 7 to + 7, with − 7 equaling a very great deal worse, zero equaling no change or improvement, and + 7 equaling a very great deal better) of patient-perceived status since the onset of treatment [42]. The GROC is valid and reliable with an MCID of two points [42], although numerous studies define major improvement as five or greater.

At each visit, the physical therapist will assess the surgical site and neurological status of the patient. The area of treatment will be examined for those patients who have received BFA. Any side effects or adverse events will be recorded in the patient’s electronic medical record and either treated or referred to an appropriate medical provider for treatment. Serious adverse events will be reported to the IRB in accordance with the approved study protocol. At the conclusion of the study, each patient’s medical record will be screened for any adverse events.

### Data analysis

An a priori power analysis was performed using G*Power, version 3.1.9.2 (Heinrich-Heine-Universitat Dusseldorf, Dusseldorf, Germany) with *α* = 0.05, *β* = 0.80, and an effect size of 0.6 (change in VAS worst pain between 24 h and 1 week post-surgery), resulting in a required sample of 90 participants. The effect size was determined through examination of data from a previous published study at the same institution [27]. To account for a potential drop-out rate of 15%, a total of 105 participants will be enrolled.

Descriptive statistics, including measures of central tendency and dispersion, will be calculated for demographic data. Frequency distributions will be estimated for categorical data. Four separate 2-by-5 mixed-model analyses of variance (ANOVA) with group as the between-subjects factor (BFA plus standard physical therapy rehabilitation versus standard physical therapy rehabilitation alone) and time as the repeated measure within-subjects factor (24 h, 48 h, 72 h, 1 week, 4 weeks) to determine the effect of BFA on pain, medication use, self-reported improvement (GROC), and mood (POMS) over the 4-week post-operative period. Alpha will be set at 0.05 for all omnibus comparisons, which are the group*time interaction, the main effect for group (fixed factor), and the main effect for time (repeated measure). Planned pairwise comparisons will be performed to examine significant main effects for group using independent *t* test, and time using paired *t* tests. Alpha for planned pairwise comparisons will be corrected using the Sidak’s correction to control for family-wise type I error. The Cohen *d* coefficient will be used to assess the effect size between pairwise comparisons. Prior to performance of the ANOVAs, all outcome measures will be assessed for normality. The appropriate non-parametric statistical tests will be used for any non-normality distributed outcomes.

When post-intervention data points are missing, data will be replaced using multiple imputation for participants who received their allocated intervention [43]. All statistical analyses will be performed with the statistical package SPSS version 26 (IBM, Chicago, IL, USA).

## Discussion

The purpose of this study is to determine differences in pain, mood, self-reported improvement, and medication use during and after a standard physical therapy rehabilitation protocol supplemented with BFA, compared to a standard physical therapy rehabilitation protocol alone, for patients following shoulder stabilization surgery. BFA may be an effective adjunct to physical therapy to reduce pain and opioid utilization in patients with acute pain [21, 27, 34]. However, the magnitude of the effect of BFA is uncertain and current studies lack blinding of outcomes assessors [21, 27, 34]. The results of this study may help determine if BFA is an effective adjunct to standard physical therapy rehabilitation post-shoulder surgery in reducing pain and opioid usage. Specifically, this study will determine if pain intensity differs between standard rehabilitation supplemented with BFA and standard rehabilitation alone during the short-term at 48 h and 72 h post-surgery, and longer-term at 1 week and 4 weeks post-surgery. Additionally, this study will determine if participants’ medication use and self-reported mood and perceptions of improvement in function differ between standard rehabilitation with and without supplemental BFA across the 4-week post-operative follow-up period.

This study is not without limitations and design constraints. The primary limitation is physical therapists and patients are not blinded to treatment groups. Many non-physiologic factors, including placebo effects, may contribute to treatment response in patients who have received BFA. We elected to forgo a sham treatment group due to the lack of feasibility in maintaining a realistic sham treatment at our institution. The most recent study of BFA that utilized a sham intervention applied the treatment while the patient was still under anesthesia (Crawford 2019). BFA represents a low-risk and low-cost alternative method of pain control when compared to opioid medications, making the potential for a placebo contribution to the response to treatment more acceptable [44, 45]. Blinding of outcomes assessors to group allocation will be conducted to minimize bias associated.

## Trial status

This study was approved by the RHC-A IRB; protocol ID number 19KACH0003, initially approved 3 September 2019, modification approved 28 October 2019. Recruitment began 25 September 2019 and will be tentatively completed in December 2022.

## Supplementary Information

**Additional file 1.**SPIRIT 2013 Checklist

**Additional file 2.** Shoulder Stabilization Rehabilitation Guidelines

**Additional file 3.** Battlefield Acupuncture Intervention

## Data Availability

The coded electronic research data for this study will be stored in Research Electronic Data Capture (REDCap), an encrypted, access-controlled, password-protected electronic data capture and management system housed on a DoD server and maintained by the Uniformed Services University of the Health Sciences Information Technology. Data from the study are available by email request to the lead author for the purpose of systematic review and meta-analysis.

## Abbreviations

ANOVA: Analysis of variance
ASP: Aiguille semi-permanente
BFA: Battlefield acupuncture
CONSORT: Consolidated Standards of Reporting Trials
DoD: Department of Defense
GROC: Global Rating of Change Scale
KACH: Keller Army Community Hospital
MCID: Minimal clinically important difference
POMS: Profile of Mood States
SPIRIT: Standard Protocol Items: Recommended for Interventional Trials
TIDieR: Template for Intervention Description and Replication
VA: Veteran’s administrations
VAS: Visual analogue scale
