# Supplementary material for: The effectiveness of battlefield acupuncture in addition to standard physical therapy treatment after shoulder surgery: a protocol for a randomized clinical trial
Source: Trials. 2020 Dec 3;21:995. doi: 10.1186/s13063-020-04909-8 (PMC7713004; doi:10.1186/s13063-020-04909-8)
Supplement: Supplementary file 2 — Additional file 2. Shoulder Stabilization Rehabilitation Guidelines [file 13063_2020_4909_MOESM2_ESM.pdf]

Keller Army Community Hospital

United States Military Academy, West Point, NY

SHOULDER RECONSTRUCTION REHABILITATION GUIDELINES

(Bankart Repair, Anterior Capsulorraphy)

PHASE I:	Generally 0 - 6 weeks post-op
**PHASE I GOALS:**	Protect the surgical repair & avoid a "stiff" shoulder
**PRECAUTIONS:**	*<u>**NO**</u> active use of arm - must <u>**ALWAYS**</u> wear sling/immobilizer, even while sleeping*
**ROM GUIDELINES:**	Avoid gaining ROM too quickly by adhering to the following:<br>• Wks 1-3: limit flexion to 90° and external rotation to 30° (neutral)<br>• Wks 4-6: limit flexion to 135° and external rotation to 50°
**SLING:**	Sling/immobilizer with abduction pillow is worn for 6 weeks per ortho/PT
**WOUND:**	Post-op dressing remains intact until post-op day #2 (~48 hours after surgery)<br>May begin showering after post-op day #2 (no need to cover incision site)<br>***Do <u>NOT</u>** submerge shoulder in tub or pool for 4 weeks*<br>Suture/staple removal @ 10-14 days per Ortho/PT<br>Begin scar massage after incision site sloughs/scar is formed
**REHABILITATION:**	*Note: Exercise prescription is dependent upon the tissue healing process and *individual* functional readiness in *all* stages. If any concerns or complications arise regarding the progress for any patient, physical therapy will contact the orthopedic surgeon.<br>Start with the following exercises:<br>**Modified Pendulum:** (May be done in the sling.) While supporting the affected arm with the unaffected hand, move the shoulder forward, backward, side to side and in clockwise and counterclockwise directions. Progress to full pendulum after 3-5 days.<br>**Supine Assisted Shoulder Flexion:** Lie on back with arm down at side and thumb pointed towards the ceiling. Use unaffected hand to grasp the wrist of the affected arm and slowly raise it until a point of mild discomfort (within ranges of motion described above).<br>**Elbow, Wrist, & Hand:** Perform elbow and wrist flexion, extension, pronation, and supination while holding the shoulder in a neutral position at side. For the hand, use a foam ball, newspaper, or theraputty to squeeze repetitively.<br>**Gentle ("Two Finger") Isometrics:** Use the unaffected hand to provide very light, pain free resistance during shoulder flexion, adduction, extension, and abduction (<u>No rotation</u>).<br>-Add gentle, pain free resistance for IR & ER starting weeks 3-4<br>**Aerobic Conditioning on Recumbent Bike:** *Sling must be worn*<br>**Scapular Retraction & Protraction:** Gently "pinch" shoulder blades together. Hold for 5 seconds and relax. Then spread shoulder blades apart. Hold for 5 seconds and relax.<br>**Shoulder AAROM exercises**: Wand, pulley, gentle towel stretch, etc.<br>**Lower Extremity Weight Lifting:** May begin at 5-6 weeks
**FOLLOW-UP:**	Physical Therapy: weekly; Ortho: ~6 wks post-op; Supervised rehab: 1-2 x per wk
**DOCUMENTATION:**	Precautions, pain level, medications and modalities<br>Observation: (incision sites) - Signs/symptoms of infection? Site healing well?<br>Neurovascular status: Distal pulses, motor and sensation intact?<br>Shoulder passive ROM (forward flexion, ER with shoulder at side)

SHOULDER LATARJET REHABILITATION GUIDELINES

PHASE I: Generally 0 - 6 weeks post-op (Immediate Post Surgical Phase)

PHASE I GOALS:
Protect the surgical repair & avoid a “stiff” shoulder
Adherence to precautions and immobilization

PRECAUTIONS:
*<u>**NO**</u> active use of arm - must <u>**ALWAYS**</u> wear sling/immobilizer, even while sleeping*
Reminder it takes 6-8 wks to form osseous union for newly reconstructed glenoid
Biceps and Coracobrachialis attachments to the coracoid need to be protected

ROM GUIDELINES:
Avoid gaining ROM too quickly by adhering to the following:

- Wks 1-3: PROM Flexion 100deg, IR 45deg, ER 25deg, Abduction Tolerance
- Wks 4-6: PROM Flexion & Abduction Tolerance, IR 45deg at 30deg ABD, ER 45deg

SLING:
Sling/immobilizer with abduction pillow is worn for 6 weeks per ortho/PT
Sleep with towel under the elbow to prevent shoulder hyperextension/wear sling

WOUND:
Post-op dressing remains intact until post-op day #2 (~48 hours after surgery)
May begin showering after post-op day #2 (no need to cover incision site)
***Do <u>NOT</u>** submerge shoulder in tub or pool for 4 weeks*
Suture/staple removal @ 10-14 days per Ortho/PT

Begin scar massage after incision site sloughs/scar is formed

REHABILITATION:
*Note: Exercise prescription is dependent upon the tissue healing process and *individual* functional readiness in *all* stages. If any concerns or complications arise regarding the progress for any patient, physical therapy will contact the orthopedic surgeon.
Start with the following exercises: (10-20 repetitions, 3-4 x daily)

~weeks 1-3

Modified Pendulum: (May be done in the sling.) While supporting the affected arm with the unaffected hand, move the shoulder forward, backward, side to side and in clockwise and counterclockwise directions. Progress to full pendulum after 3-5 days.
Elbow, Wrist, & Hand: Perform elbow and wrist flexion, extension, pronation, and supination while holding the shoulder in a neutral position at side. For the hand, use a foam ball, newspaper, or theraputty to squeeze repetitively.
Gentle (“Two Finger”) Isometrics: Use the unaffected hand to provide very light, pain free resistance during shoulder flexion, adduction, extension, and abduction (<u>No rotation</u>).
Gentle (“Two Finger”) Isometrics: add gentle, pain free resistance for IR & ER
Shoulder PROM: Do not force any painful motion.
Flexion and elevation to 100 deg
Abduction in the plane of the scapula to tolerance
Internal Rotation (IR) to 45 degrees at 30 degrees of abduction
External Rotation (ER) in the plane of the scapula from 0-25 degrees; begin at 30-40 degrees abduction

**~weeks 4-6**	**Shoulder PROM:**<br>Flexion and elevation to tolerance<br>Abduction in the plane of the scapula to tolerance<br>IR to 45 degrees at 30 degrees abduction<br>ER to 45 deg. Begin at 30-40 degrees of abduction<br>**Joint Mobilization Grade I & II:** Only if limited ROM compared to expectation for this level<br>**Scapulothoracic Mobilization:** Only if limited ROM<br>**Thoracic spine Mobilization:** Only if limited ROM<br>**Posterior Capsule stretching:** Cross body stretch, Sleeper Stretch
**FOLLOW-UP:**	Physical Therapy: weekly; Ortho: ~6 wks post-op; Supervised rehab: 2 x per wk
**DOCUMENTATION:**	Precautions, pain level, medications and modalities<br>Observation: (incision sites) - Signs/symptoms of infection? Site healing well?<br>Neurovascular status: Distal pulses, motor and sensation intact?<br>Shoulder passive ROM (forward flexion, ER with shoulder at side)
