# Supplementary material for: The effectiveness of battlefield acupuncture in addition to standard physical therapy treatment after shoulder surgery: a protocol for a randomized clinical trial
Source: Trials. 2020 Dec 3;21:995. doi: 10.1186/s13063-020-04909-8 (PMC7713004; doi:10.1186/s13063-020-04909-8)

Appendix 2 – Battlefield Acupuncture Intervention

1. Cingulate Gyrus

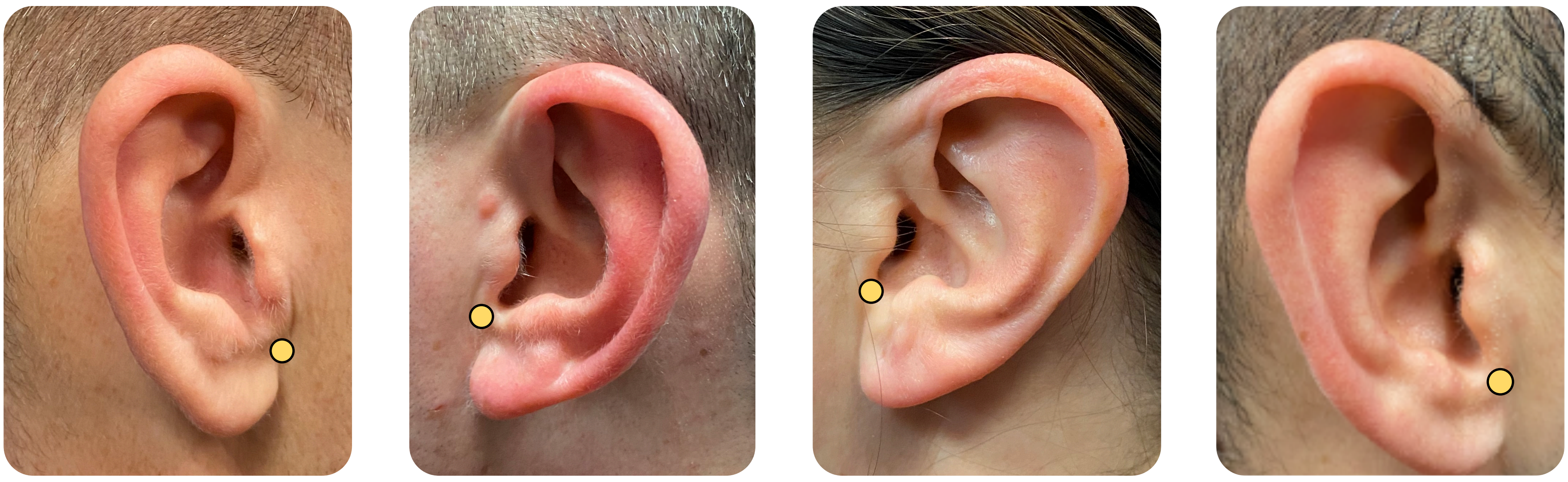

2. Thalamus

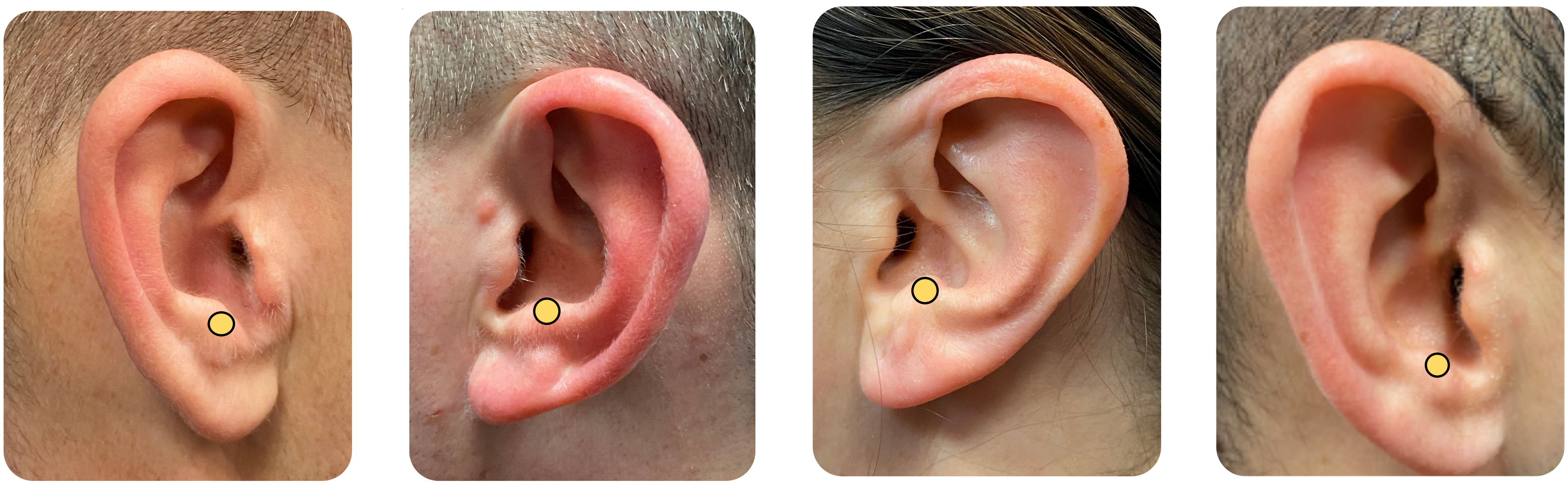

3. Omega 2

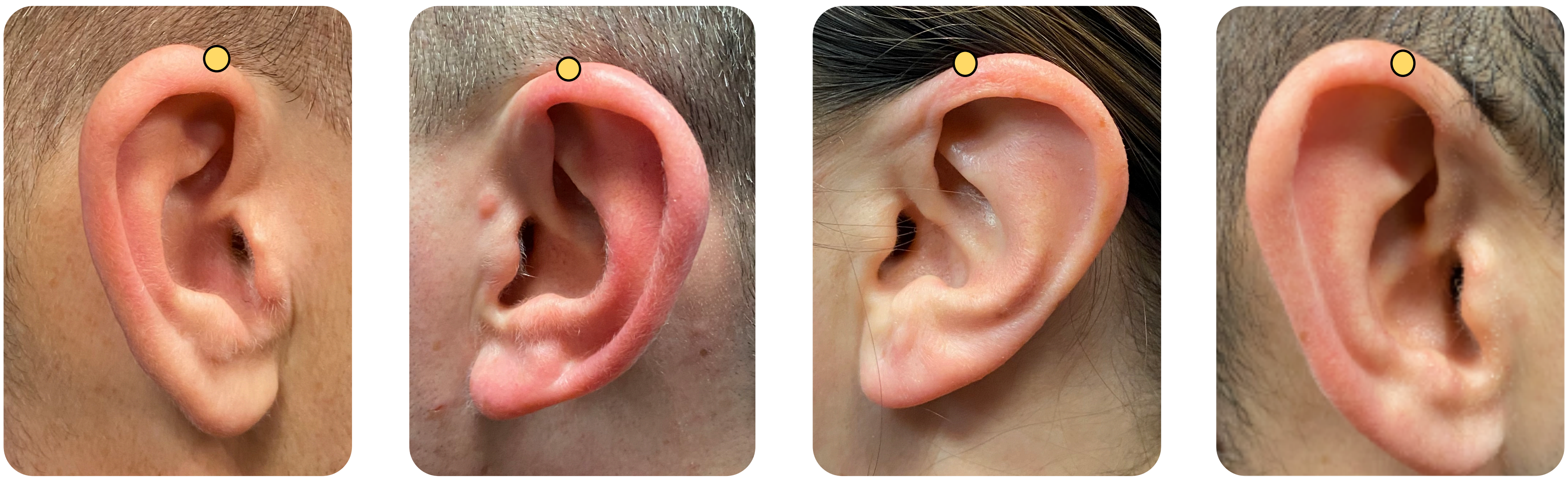

4. Point Zero

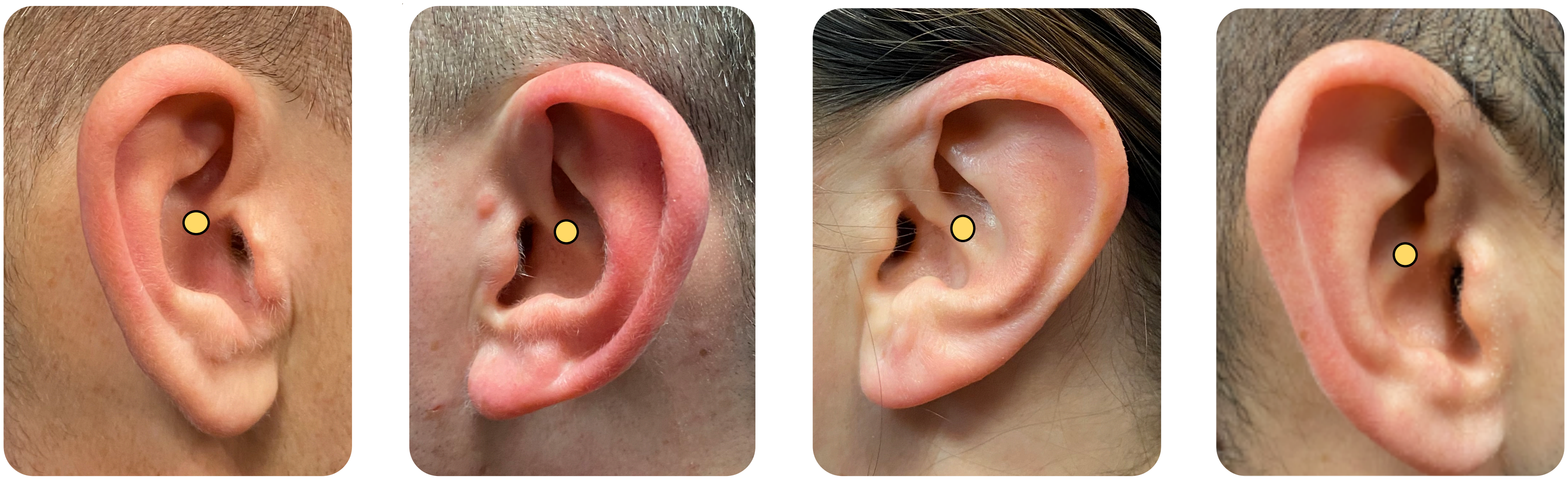

5. Shen Men

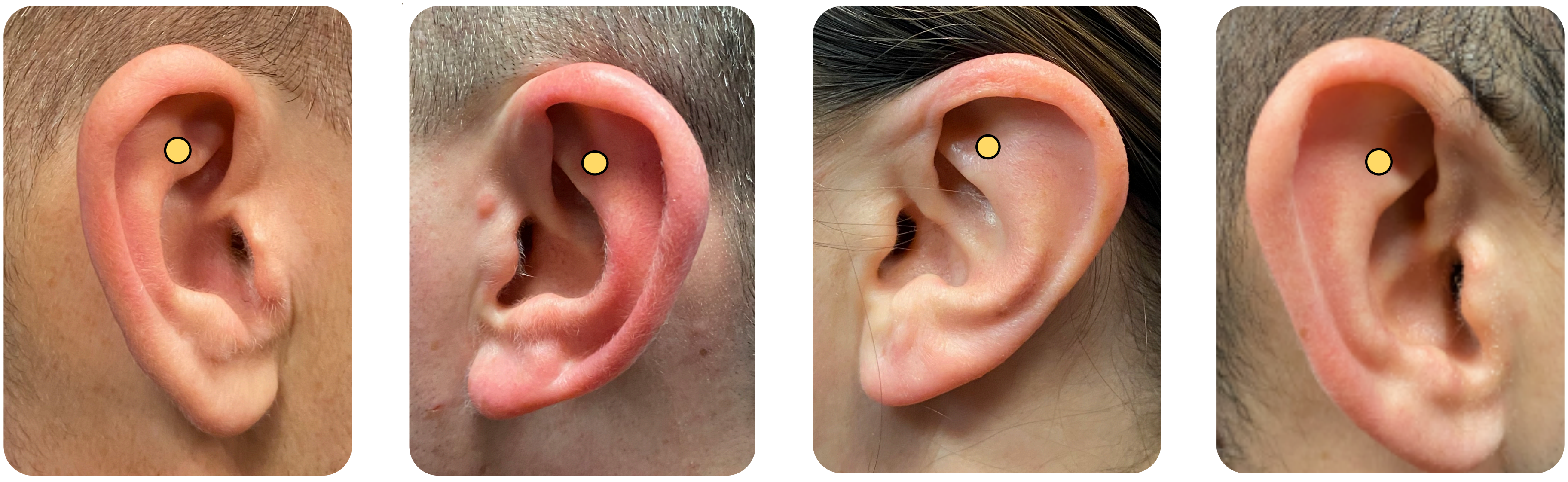

Supplement: Supplementary file 3 — Additional file 3. Battlefield Acupuncture Intervention [file 13063_2020_4909_MOESM3_ESM.pdf]
